# Association between the *IL1R2* rs2072472 polymorphism and high‐altitude pulmonary edema risk

**DOI:** 10.1002/mgg3.542

**Published:** 2019-01-22

**Authors:** Tianbo Jin, Linhao Zhu, Mei Bai, Xue He, Li Wang, Dongya Yuan, Shanqu Li, Yongjun He

**Affiliations:** ^1^ Key Laboratory of Molecular Mechanism and Intervention Research for Plateau Diseases of Tibet Autonomous Region Xianyan China; ^2^ Key Laboratory of High Altitude Environment and Genes Related to Diseases of Tibet Autonomous Region Xianyan China; ^3^ Key Laboratory for Basic Life Science Research of Tibet Autonomous Region, School of Medicine Xizang Minzu University Xianyang Shaanxi China; ^4^ Key Laboratory of Resource Biology and Biotechnology in Western China (Northwest University), Ministry of Education, School of Life Sciences Northwest University Xi’an Shaanxi China; ^5^ Medical Examination Center of Tangdu Hospital The Fourth Military Medical University Xi’an Shaanxi China

**Keywords:** case–control study, Chinese Han population, genetic polymorphism, HAPE, *IL1R2*

## Abstract

**Aim:**

High‐altitude pulmonary edema (HAPE), as a multifactorial disease, is caused by stress failure and involves both environmental and genetic factors. Study shows that *IL‐1* receptors can selectively decrease the oxygen arterial hypertension and influence the blood coagulation. So we evaluated whether genetic polymorphisms in *IL1R1* and *1L1R2* genes are associated with the risk of HAPE in Chinese Han population.

**Methods:**

Ten susceptible SNPs in the *IL1R1 *and *IL1R2 *genes were genotyped among 265 HAPE cases and 303 controls using the Agena MassARRAY platform. The associations of the SNP frequencies with HAPE were analyzed by chi‐square (χ^2^) test/Fisher's test. The genetic models were used to evaluate associations.

**Results:**

In the allele model, we found that rs2072472 was significantly associated with a 0.73‐fold decreased risk of HAPE (OR = 0.73, 95% CI = 0.55–0.97, *p* = 0.033). In the genetic model analysis, the rs2072472 in *IL1R2 *gene was associated with a 0.32‐fold decreased risk of HAPE in the codominant model, 0.67‐fold decreased risk of HAPE in the dominant model, 0.36‐fold decreasing the risk of HAPE in the recessive model, and 0.66‐fold decreased risk of HAPE in the log‐additive model, respectively. We found three candidate SNPs (rs11674595, rs4851527, and rs719250) in the *IL1R2 *gene have shown strong linkage, and none of the haplotypes was significantly associated with risk of HAPE.

**Conclusion:**

These findings suggested that *IL1R2 *polymorphisms may contribute to the protection of HAPE.

## 1 INTRODUCTION

High‐altitude pulmonary edema (HAPE), as a form of noncardiogenic pulmonary edema, is thought to be caused by an endothelial breakdown in the lungs secondary to unequal capillary pressure and its usually occurring within 2–4 days of ascent above 2,500 m (Bhagi, Srivastava, & Singh, 2014). The critical pathophysiology is an excessive rise in pulmonary vascular resistance or hypoxic pulmonary vasoconstriction (HPV) leading to increased microvascular pressures (Jensen & Vincent, 2017). Previous study showed that HAPE is associated with pulmonary hypertension and elevated capillary pressure (Maggiorini et al., 2001).

Until now, the main pathogenesis of HAPE is still not yet clear, so it is difficult to explain the disease with a single mechanism. Researches showed that HAPE was caused by a combination of genetic and environmental factors (Hotta et al., 2004). Several genetic studies have demonstrated that a genetic susceptibility may play a vital role in the development of HAPE (Luo, Zou, & Gao, 2012; MacInnis, Koehle, & Rupert, 2010; Mortimer, Patel, & Peacock, 2004). The genetic sensitivity to hypoxia has been known for years, includes severe genes (Hanaoka et al., 2009; Rong et al., 2017), such as *ACE*, *EDN1*, *ACYP2*, *RTEL1,* and *VEGF*. Genetic polymorphisms play an important role in high altitude diseases, including the HAPE risk (He et al., 2017, 2016; Mishra et al., 2012). However, genetic studies about the etiology of HAPE are still rare. Recently, Japanese scholar (Hanaoka et al., 1998) found that the HAPE human leukocyte antigen (HLA) was significantly increased compared with normal people.

Interleukin 1 (*IL1*) is a multifunctional inflammatory cytokine, which can selectively decrease the oxygen arterial hypertension and influence the blood coagulation, so some studies reported that *IL1* SNPs associate with many diseases, such as venous thrombosis (Christiansen et al., 2006; van Minkelen et al., 2007). The biological activity of the multifunctional cytokine *IL1 *is mediated by its receptors. *IL1* receptor, type 1 (*IL1R1*; OMIM: 601203) and the “decoy” receptor *IL1 *receptor type 2 (*IL1R2*; OMIM: 604512) are cytokine receptor that belong to the *IL1 *receptor family, which is an important mediator involved in many cytokine‐induced responses. Study shows that *IL1R1 *and *IL1R2 *gene regulate the cell metabolism induced by many cytokines. Moreover, epidemiological studies have been manifested that HAPE are impacted by hereditary factors.

To identify the associations between HAPE and susceptibility loci, we conducted a case–control study and identified an association between HAPE and 11 susceptible SNPs in the *IL1R1 *and *IL1R2 *gene to further clarify their potential roles in HAPE risk in the Chinese population. The study aims to evaluate a positive finding from a previous study, to provide credibility that the initial finding is valid.

## 2 MATERIALS AND METHODS

### 2.1 Ethics statement

This investigation was conducted in accordance with the ethical standards of the Declaration of Helsinki and following national and international guidelines. The venous blood was taken according to the study protocol approved by the Ethics Committee of Mental Health Center, from the Hospital of School of Medicine, Xizang Minzu University. Written informed consent was obtained from all participants after a full explanation of the study. The experimental protocol was implemented in accordance with the approved guidelines.

### 2.2 Subjects

We recruited a total of the 568 participants; 244 were diagnosed with HAPE and recruited from the Affiliated Hospital of Xizang Minzu University, China. The main inclusion criteria were based on clinical symptoms, epidemiology, and pathophysiology findings, including cough, dyspnea, cyanosis at rest, the absence of infection, and the presence of pulmonary rale (Korzeniewski, Nitsch‐Osuch, Guzek, & Juszczak, 2015). The clinical features recorded were age at diagnosis, gender, radiological results, chest sounds, body temperature, heart rate, and oxygen saturation. All HAPE patients eventually exhibited chest infiltrates consistent with pulmonary edema. Controls (*n* = 303) were healthy people selected from the same geographic region as the HAPE cases and recruited from in the outpatient departments at the hospital. All controls did not develop any symptoms and had no HAPE or related diseases after exposure to high altitude (>4,000 m) within 7 days. All subjects were Chinese Han population and resided at low altitudes <2,000 m living in northwest China. No participants used prophylactic medications, and the rate and altitude of ascent were the same among the HAPE cases and controls (altitude of the Tibetan plateau is 4,000–5,000 m).

### 2.3 SNPs selection and genotyping

Blood samples were collected in EDTA tubes and stored at −80°C after centrifugating by 17,528 *g* in 10 min. GoldMag extraction method (GoldMag Co Ltd, Xi'an, China) was used to extract genomic DNA from whole blood, and DNA concentrations were measured using a NanoDrop 2000. Ten tag SNPs in the *IL1R1 *and *IL1R2* gene were selected for our study, and these SNPs were with minor allele frequencies (MAFs) >5% in 1,000 genome (http://www.internationalgenome.org/). Agena MassARRAY Assay Design 4.0 Software was used to design a Multiplexed SNP MassEXTEND assay. SNP genotyping was performed by using Agena MassARRAY RS1000 according to the manufacturer's protocol (Gabriel, Ziaugra, & Tabbaa, 2009). Agena Typer 4.0 software was used for data management and analysis.

### 2.4 Statistical analysis

Statistical analyses were performed using SPSS version 19.0 for Windows (SPSS, Chicago, IL, USA) and SNPstats software platform (https://www.snpstats.net/). Each SNP frequency in the control subjects was tested for deviation from Hardy–Weinberg equilibrium (HWE) by the Fisher's test. The genotype and allele frequencies in patients and controls were calculated by the χ2 test (Adamec, 1964). Odds ratio (OR) values and 95% confidence intervals (CIs) measured risk allele effect size using unconditional logistic regression analysis with adjustments for age and gender (Bland & Altman, 2000). Finally, the Haploview was used to construct haplotype and genetic association at significant polymorphism loci and to estimate the pairwise linkage disequilibrium (LD) (Barrett, Fry, Maller, & Daly, 2005), haplotype software (version4.2), and SHEsis software platform (http://analysis.bio-x.cn/myAnalysis.php), and genetic association at polymorphism loci (Shi & He, 2005). All *p *values presented in this study are two‐sided test; *p < *0.05 indicates a statistically significant difference. The statistical methods of this study were all conducted by reference to the methods of Dai et al (Dai et al., 2016; Zhou et al., 2018).

## 3 RESULT

### 3.1 Characteristics of the participants

This study involved 565 subjects, including 265 patients (244 males and 21 females; age at diagnosis: 32.6 ± 10.7 years) and 303 healthy controls (289 males and 14 females; age: 36.2 ± 4.5 years). The HAPE cases and controls were matched by sex, but there was a significant difference in age between HAPE cases and controls (*p < *0.001) (Table 1).

**Table 1:** General characteristics of this study population

Variable	Cases	%	Controls	%	*p* Value
	(*n* = 265)		(*n* = 303)		
Gender					
Male	244	45.80	289	54.20	>0.05^a^
Female	21	60.00	14	40.00	
Age, year (mean ± *SD*)	32.6 ± 10.7		36.2 ± 4.5		<0.001^b^

a *P* values were calculated by Student's *t* tests.

b *P* values were calculated from two‐sided chi‐square tests.

### 3.2 The associations between IL1R1 and IL1R1 SNPs and HAPE

Table 2 summarizes the basic information of candidate SNPs in our study, such as chromosomal position, gene, allele, HWE test results, and MAF, 95% CI, and the *p* value of allele. Three SNPs (rs102631625, rs102641201, and rs102726661) were excluded for significant deviation from HWE (*p < *0.05). We used the chi‐squared test to assess the risk of gene polymorphism in the allele model and found that rs2072472 was significantly associated with a decreased risk of HAPE (OR = 0.73, 95% CI = 0.55–0.97, *p* = 0.033). However, the other seven SNPs (rs102610992, rs102622376, rs102623718, rs102717337, rs102758116, rs102769302, and rs102792760) had no significant association with HAPE risk. There is no statistically significant association between allele and HAPE risk by Bonferroni correction.

**Table 2:** Allele frequencies in cases and controls and odds ratio estimates for HAPE risk

Position	Gene (s)	Locus	Alleles (A/B)	MAF		HWE *p*	OR (95% CI)	*p* Value
				Case	Control			
rs11674595	*IL1R2*	2q11.2	C/T	0.184	0.213	0.490	0.84 (0.62–1.12)	0.237
rs4851527	*IL1R2*	2q11.2	A/G	0.257	0.266	0.378	1.95 (0.73–1.24)	0.728
rs719250	*IL1R2*	2q11.2	T/C	0.34	0.318	0.236	1.10 (0.86–1.41)	0.449
rs3218896	*IL1R2*	2q11.2	C/T	0.16	0.163	0.019	0.98 (0.71–1.34)	0.891
rs3218977	*IL1R2*	2q11.2	G/A	0.272	0.264	0.018	1.04 (0.80–1.35)	0.771
rs2072472	*IL1R2*	2q11.2	G/A	0.181	0.233	0.749	0.73 (0.55–0.97)	**0.033** *
rs10490571	*IL1R1*	2q12.1	T/C	0.197	0.223	0.508	0.86 (0.64–1.14)	0.288
rs956730	*IL1R1*	2q12.1	A/G	0.268	0.256	0.292	1.06 (0.82–1.39)	0.642
rs3917225	*IL1R1*	2q12.1	T/C	0.404	0.422	0.724	0.92 (0.73–1.17)	0.524
rs3917318	*IL1R1*	2q12.1	G/A	0.475	0.436	0.415	1.17 (0.93–0.48)	0.179

Notes

CI: confidence interval; HWE: Hardy–Weinberg equilibrium; MAF: minor allele frequency; *p‐*_HWE_ < 0.01 indicates imbalance; OR: odds ratio; SNP: single‐nucleotide polymorphism.

* *p* < 0.05 indicates statistical significance.

### 3.3 Associations between genotype frequencies and HAPE

As shown in Table 3, we found the rs2072472 in the *IL1R2* gene was associated with a 0.72‐fold decreased risk of HAPE in the log‐additive model (OR = 0.72, 95% CI = 0.53–0.97, *p* = 0.029). However, when adjusted by gender and age, we found that the rs2072472 in *IL1R2 *gene was associated with a 1.64‐fold increase the risk of HAPE in the codominant model (OR = 0.32, 95% CI = 0.12–0.87, *p* = 0.022 for the “G/G” genotype), 0.67‐fold decreased risk of HAPE in the dominant model (OR = 0.67, 95% CI = 0.47–0.96, *p = *0.026 for the “A/G ‐ G/G” genotype), 0.67‐fold decreased risk of HAPE in the recessive model (OR = 0.36, 95% CI = 0.13–0.97, *p* = 0.033 for the “G/G” genotype), and 0.66‐fold decreased risk of HAPE in the log‐additive model (OR = 0.66, 95% CI = 0.49–0.90, *p* = 0.009), respectively.

**Table 3:** Relationships between *IL1R2* polymorphism and HAPE risk

SNP	Model	Genotype	Control	Case	Before adjusted		After adjusted		AIC	BIC
					OR (95% CI)	*p* a	OR (95% CI)	*p* b		
rs2072472	Codominant	A/A	177 (58.4%)	175 (66%)	1	0.074	1	**0.022** *	754.9	776.6
		A/G	111 (36.6%)	84 (31.7%)	0.77 (0.54–1.09)		0.72 (0.50–1.04)			
		G/G	15 (5%)	6 (2.3%)	0.40 (0.15–1.07)		**0.32 (0.12–0.87)**			
	Dominant	A/A	177 (58.4%)	175 (66%)	1	0.062	1	**0.026** *	755.6	772.9
		A/G‐G/G	126 (41.6%)	90 (34%)	0.72 (0.51–1.02)		**0.67 (0.47–0.96)**			
	Recessive	A/A‐A/G	288 (95%)	259 (97.7%)	1	0.084	1	**0.033** *	755.9	773.3
		G/G	15 (5%)	6 (2.3%)	0.44 (0.17–1.16)		**0.36 (0.13–0.97)**			
	Log‐additive	–	–	–	**0.72 (0.53–0.97)**	**0.029** *	**0.66 (0.49–0.90)**	**0.009** *	753.6	771

Notes

OR: odds ratio; SNP: single‐nucleotide polymorphism; 95% CI: 95% confidence interval.

^a^ *p* values were calculated from unconditional logistic regression analysis. ^b^ *p *values were adjusted by age and gender. ^*^ *p < *0.05 indicates statistical significance.

### 3.4 Associations between haplotype analyses and HAPE risk

**Figure 1:**
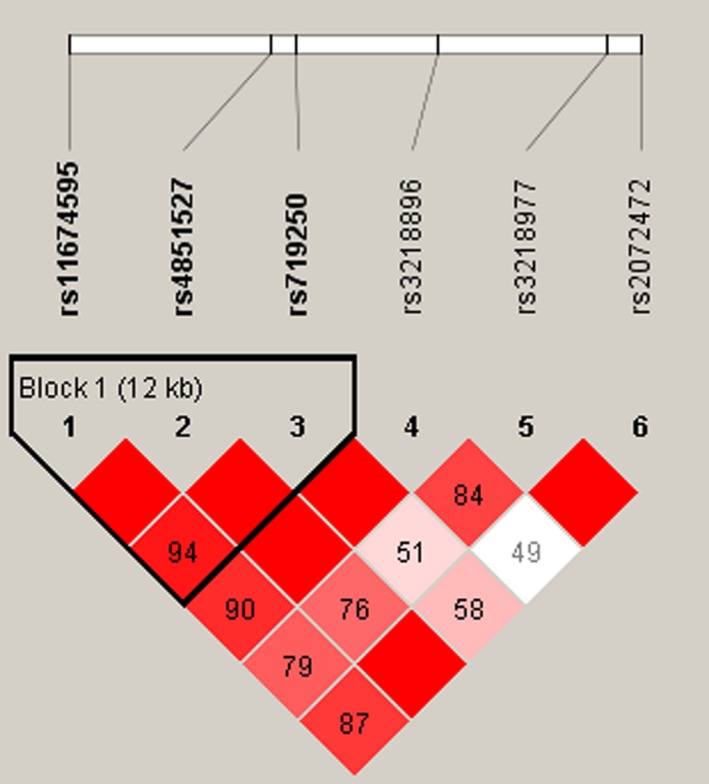
Linkage disequilibrium (LD) plots containing four SNPs from *IL1R2*

Linkage disequilibrium and haplotype analyses of the SNPs in the case and control samples were further studied. In order to assess the association between haplotypes and HAPE risk, a Wald test was performed using an unconditional multivariate regression analysis. Although the three candidate SNPs (rs11674595, rs4851527, and rs719250) in the *IL1R2 *gene have shown strong linkage (Figure 1), the result for the *IL1R2 *haplotype was not found to be associated with a risk of HAPE, because the *p *value has no statistical difference (Table 4). In addition, we have not found any association between *IL1R1 *haplotype and the risk of HAPE.

**Table 4:** Haplotype analysis results in this study

	Haplotypes			Freq	Without adjusted		With adjusted	
	rs11674595	rs4851527	rs719250		OR (95% CI)	*p* Value	OR (95% CI)	*p* a Value
1	T	G	T	0.325	1	–	1	–
2	T	A	C	0.2614	0.92 (0.68–1.25)	0.6	0.97 (0.70–1.33)	0.83
3	T	G	C	0.2129	1.03 (0.74–1.45)	0.85	1.10 (0.77–1.55)	0.61
4	C	G	C	0.1973	0.80 (0.57–1.13)	0.21	0.75 (0.53–1.06)	0.11

Notes

OR: odds ratio; *p* ^a^: adjusted by gender and age; SNP: single‐nucleotide polymorphism; 95% CI: 95% confidence interval.

## 4 DISCUSSION

High‐altitude pulmonary edema is mainly due to exposure to the oxygen‐depleted environment and triggers the onset of a range of physiological and biochemical reactions. Nowadays, due to the increase in skiing, trekking, and climbing tours, the HAPE mortality rates have reached 50% (Bartsch & Swenson, 2013). In HAPE, the occurrence rate of HLA was significantly increased. One study indicates that the Han migrants were relative easier to have HAPE and the reason might related to their different genetic background (Luo et al., 2012). Several studies have shown that the genetic SNPs have significant association with HAPE risk (Aggarwal et al., 2015; Kobayashi et al., 2013; Pandey, Mohammad, Singh, & Qadar Pasha, 2015).

In the present case–control study, we investigated the associations between 11 SNPs in *IL1R1* and *IL1R2* gene risk of HAPE. We demonstrated that *IL1R2* genetic polymorphisms are associated with HAPE risk in Chinese Han population. Our results show that the rs2072472 in the *IL1R2* gene was associated with protection from HAPE. These results suggest that the polymorphism of *IL1R2* gene may play an important role in the risk of HAPE in the Han Chinese population.

Interleukin 1 is important in promoting coagulation by down‐regulating the expression of thrombomodulin and endothelial cell protein C receptor (Esmon, 1994). Furthermore, it can selectively decrease the oxygen arterial hypertension and influence the blood coagulation. The biological activity of the multifunctional cytokine *IL1* is mediated by its receptors. *IL1R1* and *IL1R2* are cytokine receptors that belong to the *IL1* receptor family, which is an important mediator involved in many cytokine‐induced responses. Some studies reported that *IL1R1 *and *IL1R2 *SNPs are associated with venous thrombosis (Christiansen et al., 2006; van Minkelen et al., 2007) and immune and inflammatory disease (Latiano et al., 2013; Xie et al., 2017). *IL1R1*, as a protein‐coding gene, is located in a cluster of related cytokine receptor genes on chromosome 2q12, which belongs to the *IL1* receptor family and encodes a cytokine receptor (Vasilyev, Silkov, & Sennikov, 2015). Although previous studies have reported that *IL1R1* gene is associated with an increased risk of many diseases, in this study *IL1R1 *is not associated with the risk of HAPE, this may be due to the relatively small sample size. *IL1R2, *as a decoy receptor, is located on 2q11.2 in the human gene. This decoy receptor has no singling properties, it can neutralize the agonist effects by preventing IL1 from reaching signal IL1R1, which restricting the cytokine's biological effects. (Khoufache et al., 2012). Many studies reported that the *IL1R2 *as a protected factor can decrease the risk of many diseases, such as IgA nephropathy (Xie et al., 2017), Arthritis (Shimizu et al., 2015), and atherosclerosis (Pou et al., 2011). However, there were few researches about the IL1R2 genetic polymorphisms in previous studies, and no studies have been reported the genetic polymorphism of IL1R2 and HAPE risk. For this study, the rs2072472 in *IL1R2* showed a decreased risk in HAPE. Hence, *IL1R2 *gene may play an important function in affecting HAPE. But the distinct role of *IL1R2*, especially in HAPE, remains unknown and is worth our further research.

To sum up, we provide new evidence for the association between *IL1R1* and *IL1R2* variant and HAPE risk in Han Chinese population for the first time, which may provide new data to facilitate earlier diagnosis and promote early prevention, and shed light on the new candidate genes and new ideas for the study. Nevertheless, there are limitations that need to be noticed. Our current research is fundamental; further functional studies and larger population‐based prospective studies are required in order to understand the genetic factors underlying HAPE.

## CONFLICT OF INTEREST

The authors have no conflicts of interest to disclose.
